# Repurposing antimalarial artesunate for the prophylactic treatment of depression: Evidence from preclinical research

**DOI:** 10.1002/brb3.2833

**Published:** 2022-12-27

**Authors:** Shihao Huang, Ewa Galaj, Jinfeng Wang, Yi Guo, Shuang Wang, Mengxu Shi, Xueyong Yin, Keyao Liu, Yixiao Luo, Li Meng, Haishui Shi

**Affiliations:** ^1^ National Institute on Drug Dependence and Beijing Key Laboratory of Drug Dependence Peking University Beijing China; ^2^ Department of Psychological and Brain Sciences Colgate University Hamilton New York USA; ^3^ Department of Obstetrics and Gynecology The No.1 Hospital of Yongnian District Handan City Handan China; ^4^ Neuroscience Research Center Institute of Medical and Health Science of HeBMU Hebei Medical University Shijiazhuang China; ^5^ Hebei Key laboratory of Neurophysiology Hebei Medicinal University Shijiazhuang China; ^6^ Department of Biomedical Engineering University of North Carolina Chapel Hill North Carolina USA; ^7^ Hunan Province People's Hospital The First‐affiliated Hospital of Hunan Normal University Changsha China

**Keywords:** artesunate, depression, inflammation, lipopolysaccharide, mouse

## Abstract

**Introduction:**

Several studies have linked inflammation and oxidative stress with the pathogenesis of depression. Artesunate is a commonly used medication to treat malaria and has been shown to produce antioxidant, anti‐inflammatory, and immunomodulatory effects. However, its prophylactic effects on depression and depression‐related brain pathology are unknown.

**Methods:**

In Experiment 1, using a PC12 cell line, we investigated whether artesunate can prevent hydrogen peroxide (H_2_O_2_)‐induced oxidative injury that mimics oxidative stress commonly observed in the depressed brain. Next, using lipopolysaccharide (LPS)‐induced mouse model of depression, we investigated whether artesunate can prevent behavioral deficits observed in the open field test, novelty‐suppressed feeding test, sucrose preference test, forced swimming test, and tail suspension procedure.

**Results:**

We found that artesunate significantly prevented a H_2_O_2_‐induced reduction in PC12 cell activity, suggesting its antioxidant potential. We also found that mice pretreated with artesunate (5, 15 mg/kg) intraperitoneally (i.p.) prior to the LPS (.8 mg/kg, i.p.) treatment showed fewer and less severe depression‐ and anxiety‐like behaviors than the LPS‐treated control mice.

**Conclusion:**

Our findings indicate that artesunate produces antioxidant effect, as well as antidepressant and anxiolytic effects. Importantly, our findings first demonstrate that artesunate can prevent LPS‐induced depression‐ and anxiety‐like symptoms, strongly suggesting its prophylactic potential in the treatment of depression and, perhaps, other psychiatric disorders associated with inflammation and oxidative stress.

## Graphical abstract

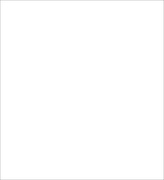

## 1 INTRODUCTION

Major depression is the leading cause of mental disability, impacting over 300 million people worldwide (Disease et al., 2018). Available tricyclic medications and selective serotonin inhibitors for depression have a slow onset and can cause many unpleasant side effects including nausea, weight gain, sleep problems, and sexual dysfunction (Ferguson, 2001). These adverse effects, in addition to limited efficacy of antidepressants, often decrease compliance and delayed recovery. Thus, new therapeutic or prophylactic approaches are desperately needed.

Macrophage‐induced inflammation has been identified as a prominent biological risk factor in the pathogenesis of depression (Porter & O'Connor, 2022; Smith, 1991) and many clinical and animal studies highlight strong associations between inflammation and behavioral symptoms of depression (Kitaoka, 2022; Kohler et al., 2016; Lindqvist et al., 2017; Miller et al., 2009; Zhang et al., 2019). For example, high concentration of proinflammatory cytokines TNF‐alpha and IL‐6 were found in patients with major depression (Beurel et al., 2020; Dowlati et al., 2010) and clinical trials have indicated beneficial effects of anti‐inflammatory medications on major depression symptoms (Akhondzadeh et al., 2009; Muller et al., 2006). In rodents, systemic lipopolysaccharides (LPS) are known to increase inflammatory responses, leading to significant depression‐ and anxiety‐like behaviors, which imitate depression in humans (Luo et al., 2021; Zhao et al., 2019). Given that depression is associated with inflammation, it is conceivable that anti‐inflammatory drugs might have prophylactic potential for the treatment of depression.

Artesunate is an antimalarial drug that is recommended by the World Health Organization as a standard treatment for cerebral and other types of malaria (WH, 2015) and has recently been approved by the United States (US Food and Drug Administration, 2020). This antimalarial drug is known for its safety profile and high efficacy, and recently, it has been shown to prevent lipopolysaccharide (LPS)‐induced inflammation by interfering with NF‐кB and p38 mitogen‐activated protein kinase (MAPK) signaling (Mancuso et al., 2021; Okorji & Olajide, 2014). Artesunate has the ability to restore spatial learning in rats with hepatic encephalopathy (Wu et al., 2016), a pathological condition caused by a build‐up of toxins and high levels of inflammation in the brain. Despite studies showing a neuroprotective role of artesunate (Zhu et al., 2018), to date, it is unknown whether this antimalarial drug produces anti‐depressant effects. In the present study, we aimed to investigate whether artesunate can prevent hydrogen peroxide (H_2_O_2_)‐induced oxidative injury that mimics oxidative stress commonly observed in the depressed brain and whether artesunate can block behavioral deficits in mice exposed to the LPS treatment. We assessed prophylactic effects of artesunate in a battery of behavioral tests, including the open field test (OFT), novelty‐suppressed feeding (NSF) test, sucrose preference test, forced swimming test (FST), and tail suspension procedure.

## 2 METHODS

### 2.1 Animals

Male ICR mice (aged 7 weeks old upon arrival) were purchased from the Beijing Vital River Laboratory Animal Technology Co. Ltd., China. Mice were kept in a climate‐controlled environment with 23 ± 2°C temperature and 40%–60% humidity and with a 12 h reverse light/dark cycle (light on at 8:00 p.m., off at 8:00 a.m.). Mice had free access to food and water all the time, except during experimentation. The housing conditions and animal care were consistent with the Guide for the Care and Use of Laboratory Animals. All experimental procedures were approved by the Local Committee on Animal Care and Use and Protection of the Hebei Medical University.

### 2.2 Drugs

In cell culture, artesunate (CAS‐88495‐63‐0), purchased from Yuanye Bio‐Technology (Shanghai, China), was dissolved in dimethyl sulfoxide (DMSO) at a concentration of 0.1 M. Aliquots were stored in −20°C and diluted in DMSO before use. In all experiments, cells were treated with artesunate (.25−2 μM) in the absence or presence of H_2_O_2_ (150 μM). Fetal bovine serum (FBS) was purchased from ScienCell. High‐glucose Dulbecco's Modified Eagle's Medium (DMEM) medium was purchased from GEL Life Science (Logan, Utah, USA). All chemicals and reagents were of analytical grade.

For behavioral testing, artesunate and LPS (Sigma‐Rich Trading Co. Ltd., Shanghai, China) were freshly dissolved in 0.9% physiological saline and were prepared on the day of injection. The doses of artesunate/LPS were chosen based on previous studies and kept at a volume of 5 ml/kg of body weight (Lu et al., 2018). Mice were injected with LPS at the dose of 0 or 0.8 mg/kg, i.p. 24 h prior to behavioral tests. To investigate a potential prophylactic role of artesunate on LPS‐induced behavioral abnormalities, mice had been pretreated with artesunate (0, 5, 15 mg/kg, i.p.) twice a day for 9 consecutive days prior to the LPS treatment (day 10). From day 11, mice were given additional injections of artesunate (0, 5, 15 mg/kg) 30 min prior to each behavioral test.

### 2.3 Behavioral tests

#### 2.3.1 Open field test

An OFT was conducted in black boxes (40 × 40 × 35 cm) with the central zone on the floor (20 × 20 cm). The total distance traveled and time spent in the center zone were recorded for 5 min and analyzed by the SMART v3.0.02 software. Two experimenters blinded to the conditions scored the time spent in the center zone and the averaged scores from the two observations were used. This procedure was based on our previous studies (Gong et al., 2017).

#### 2.3.2 Tail suspension test

A tail suspension test (TST) measures a depression‐like behavior and was performed according to our published methods (Gao et al., 2016; Wu et al., 2016). Mice were suspended 50 cm above the floor using an adhesive tape placed approximately 1 cm from the tip of the tail. The test lasted 6 min and the latency to immobility within the first 2 min and the immobility time during the final 4 min of the test were measured. Immobility, an index of depression‐like behavior, was defined as the time during which the animal was hanging passively and motionlessly. Two trained observers blinded to the conditions scored the average immobility time (s) during 6‐min TST.

#### 2.3.3 Forced swimming test

A FST was carried out as previously described (Gao et al., 2016; Wu et al., 2016) using a plastic cylinder (12 cm diameter × 30 cm height) filled with 23–25°C water to 15 cm height. Mice were placed in water for the 6 min FST. Their immobility latency during the first 2 min and immobility time during the last 4 min were measured. Immobility, an index of depression‐like behavior, was defined as the absence of movement except motions required to maintain the animal's head above the water. Observers blinded to the treatment conditions measured the latency and immobility time.

#### 2.3.4 Novelty‐suppressed feeding test

NSF was assessed according to our published procedures (Gong et al., 2017; Wu et al., 2016). Mice were subjected to 24 h food deprivation. Next day, each mouse was placed in a corner of the novel box (40 × 40 × 35 cm) with food chow positioned in the brightly lit center of the box. The latency to eat was measured in mice. If mice did not initiate food consumption within 300 s, their latency was marked as 300 s. Mice were then transferred to their home cages and their food consumption was measured for the next 10 min. Two observers blinded to the treatment conditions measured the latency to eat and a total food intake for each mouse to assess the anxiety‐like behavior and appetite.

#### 2.3.5 Sucrose preference test

In this experiment, mice were subjected to a two‐bottle sucrose preference test to measure their anhedonia‐like responses. The SPT was carried out according to our published procedure (Gong et al., 2017; Wu et al., 2016). Mice were pre‐exposed to 1% sucrose for 48 h in their home cages. Next, mice were subjected to 24 h water deprivation. On the SPT day, two bottles—one with 1% sucrose water and the other with water—were provided for each mouse for total 24 h. The volume and weight of both sucrose water and ordinary water were recorded prior and post SPT to assess consumption. Sucrose preference calculated as sucrose water intake/(sucrose water intake + ordinary water intake) *100% was an indicative of depressive‐like behavior.

### 2.4 Experimental design

#### 2.4.1 The effect of artesunate on H_2_O_2_ ‐induced cellular injury

We investigated the effects of artesunate on oxidative stress using the MTS cell proliferation assay. The method of cell culture was carried out according to the literature (Okorji et al., 2016). Rat pheochromocytoma (PC12 cells) was maintained in DMEM containing 10% FBS. PC12 cells were cultured at 37°C in 5% CO_2_ in a humidified atmosphere. Cells were plated on polystyrene culture dishes, and 24 h after incubation, they were fed with fresh medium for treatment. PC12 cells were plated at a density of 1.0×10^5^ cells/well in 96‐well culture plates and treated with artesunate (0.25−2 μM) in the absence or presence of H_2_O_2_ (150 μM). The optical density in the culture media was measured by the MTS assay using CellTiter 96Ò AQueous One Solution cell proliferation kit (Promega, Madison, WI, USA) according to the manufacturer's protocol.

#### 2.4.2 The effect of pretreatment with artesunate on the depressive‐like and anxiety‐like behaviors

After habituation for 5 days, mice were treated with one of the artesunate doses (0, 5, 15 mg/kg, i.p.) twice a day for 8 days. To evaluate the antidepressant and anxiolytic‐like activities of artesunate, on day 5 and onward, behavioral tests, including OFT, TST, FST, and NSF were conducted 30 min after artesunate administration.

#### 2.4.3 The effect of artesunate on LPS‐induced deficits observed in the depressive‐like and anxiety‐like tests

Mice were pretreated with one of the artesunate doses (0, 5, 15 mg/kg, i.p.) twice a day for 9 days. On day 10, mice were injected with LPS at a dose of 0 or 0.8 mg/kg, i.p. To investigate the effect of artesunate on LPS‐induced deficits, on day 11 and onward, 30 min prior to the behavioral tests, mice were given an additional injection of artesunate (0, 5 or 15). Behavioral tests, including OFT, TST, FST, NSF, and SPT, were conducted as in the above description.

### 2.5 Data analysis

All data were expressed as the mean ± SEM. Separate one‐way Analyses of Variance (ANOVAs) were employed to analyze the behavioral tests data and cell culture data, followed by post hoc Tukey tests. Dependent variables were: in the OFT, total distance traveled and time spent in the center zone; in the NFT, latency to eat and a total food intake; in the SPT, sucrose consumption; in the TST, latency to immobility and the immobility time; and in the FST, immobility latency and immobility time. *ps* < .05 were considered statistically significant.

## 3 RESULTS

### 3.1 Artesunate produced protective effects in PC12 cells exposed to H_2_O_2_

**FIGURE 1:**
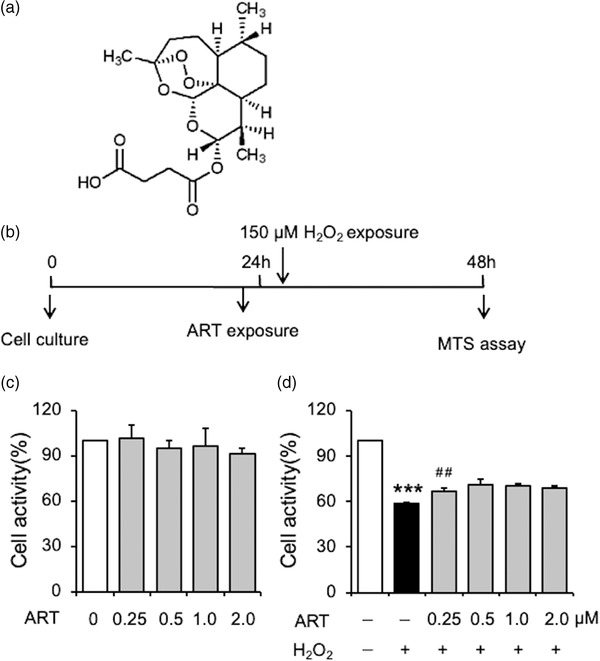
Artesunate 0.25–2 μM reversed the decreased cell activity induced by H2O2. (a) Chemical structure of artesunate. (b) Experimental procedure. The PC12 cells were treated with artesunate (0, 0.25, 0.5 1.0, 2.0 μM) and co‐treated with 150 μM H_2_O_2_ for 24 h. (c) The PC12 cells were treated with artesunate without H_2_O_2_. (d) The PC12 cells were treated with artesunate and co‐treated with H_2_O_2_. ***p < .001 as compared to Control group, ^##^ *p* < .01 versus H_2_O_2_ group, *n* = 5 per group

To determine whether artesunate (Figure 1a) can prevent H_2_O_2_‐induced cellular injury, we treated PC12 cells with artesunate (0, 0.25, 0.5, 1.0, and 2.0 μM) in the presence or absence of H_2_O_2_ (150 μM) for 24 h and analyzed cell viability using the MTS assay, as indicated in Figure 1b. Subsequent determination of cell viability showed that artesunate on its own did not decrease cellular proliferation (*F*_4,24_ = .332, *p* = .853, *n* = 5; Figure 1c). A one‐way ANOVA analysis showed that artesunate exerted significantly protective effects against H_2_O_2_‐induced cellular injury (*F*_5,29_ = 53.117, *p* < .001, Figure 1d). Post hoc analysis indicated that .25 μM artesunate significantly reversed the H_2_O_2_‐induced reduction in cell proliferation (*p* = .005). These results indicate that pretreatment with artesunate exerts protective effects against H_2_O_2_‐induced cellular injury.

### 3.2 Artesunate exerted antidepressant‐ and anxiolytic‐like effects

**FIGURE 2:**
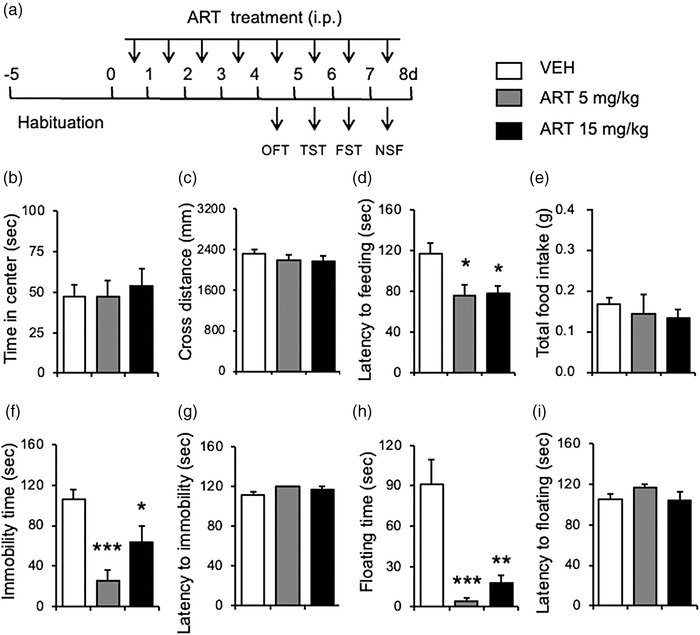
Artesunate exerted antidepressant‐ and anxiolytic‐like effects. (a) Experimental procedure. After 5‐day adaptation period, the mice were injected with vehicle, artesunate 5 mg/kg, 10 mg/kg twice daily for 8 days. From 5th day, behavioral test was conducted. Pretreatment with artesunate had no effect on time spent in the center zone (b) and crossing distance (c) in the OFT, decreased latency time to feeding (d) without affecting the total feeding (e) in the NSF, decreased immobility time (f) without affecting the latency to immobility time (g), and decreased floating time (h) without affecting the latency to floating time (i). *p < .05, **p < .01, ***p < .001 as compared to VEH group. *n* = 8–11 per group.

Figure 2a indicates a schematic timeline of the treatment and behavioral assays. Mice were randomly divided into three groups (*n* = 8–11 per group): vehicle (VEH group), 5 mg/kg artesunate‐treated group, and 15 mg/kg artesunate‐treated group. Mice were injected with saline or artesunate (5, 15 mg/kg, i.p.) twice daily for 8 consecutive days. On day 5 and later, mice were subjected to behavioral tests. Thirty minutes before each behavioral test, mice received injections of artesunate, as indicated in Figure 2a.

Figure 2b shows that during the OFT, mice treated with artesunate (5, 15 mg/kg) spent a similar amount of time in the center zone as vehicle‐treated mice. A one‐way ANOVA confirmed this observation and revealed no significant difference in the time spent in the center zone between groups (*F*_2,26_ = .154, *p* = .858, Figure 2b) and total distance travelled during the test (*F*_2,26_ = .875, *p* = .429, Figure 2c). The results indicate that artesunate treatment failed to alter the open field locomotor activity.

In the NSF test, the artesunate‐treated (5, 15 mg/kg) mice showed suppressed eating behavior in a novel environment, as compared to the control. Mice treated with artesunate showed shorter latency to feeding than the vehicle‐treated group. This observation was confirmed by a one‐way ANOVA that revealed a significant difference in the latency to feeding among three groups (*F*_2,26_ = 5.235, *p* = .016, Figure 2d). A post hoc Tukey's test showed that both the 5 mg/kg (*p* = .024) and 15 mg/kg (*p* = .031) of artesunate prolonged the immobility latency as compared to the vehicle‐treated group. There were no significant effects of artesunate on total food intake (*F*_2,26_ = .329, *p* = .723, Figure 2e). The results indicate that artesunate treatment significantly reduced the anxiety‐like behaviors.

In the TST, mice treated with artesunate (5, 15 mg/kg) showed significant greater immobility than the vehicle‐treated mice. Reduced immobility (time being motionless) was observed in mice treated with artesunate (Figure 2f). A one‐way ANOVA of the TST data showed there was a significant difference in the total immobility time among groups (*F*_2,26_ = 12.113, *p* < .001, Figure 2f). The latency to immobility time was not different between groups (*F*_2,26_ = 2.573, *p* = .097, Figure 2g). A post hoc Tukey's test showed that both 5 mg/kg (*p* < .001) and 15 mg/kg (*p* = .031) of artesunate significantly decreased the immobility time.

In the FST, for mice treated with artesunate (5, 15 mg/kg), their immobility time was drastically reduced as compared to the vehicle‐treated mice (Figure 2h). A one‐way ANOVA revealed a significant artesunate treatment effect in the floating time (*F*_2,26_ = 13.962, *p* < .001, Figure 2h) but not the latency to floating (*F*_2,26_ = 1.530, *p* = .235, Figure 2i). A post hoc Tukey's test showed that both 5 mg/kg (*p*< .001) and 15 mg/kg (*p* = .031) of artesunate significantly decreased the floating time. Together, these results suggest that artesunate exerted antidepressant‐ and anxiolytic‐like effects in mice.

### 3.3 Artesunate pretreatment significantly blocked LPS‐induced depression‐ and anxiety‐like behaviors

**FIGURE 3:**
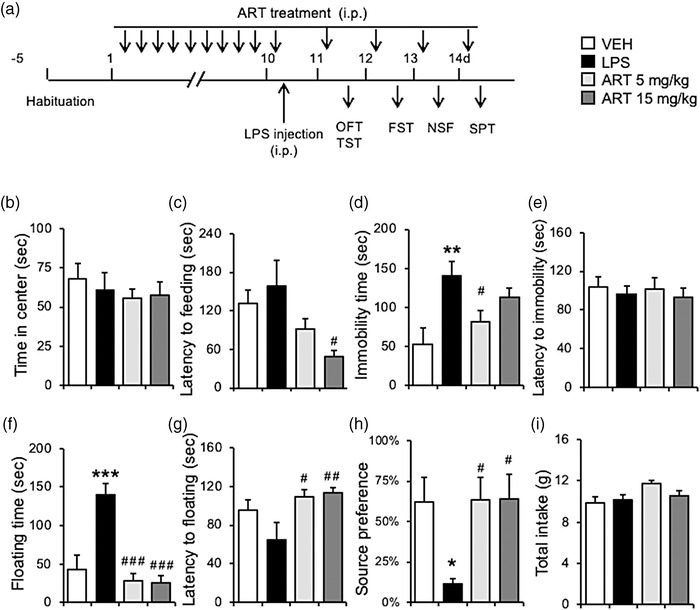
Artesunate pretreatment significantly blocked LPS‐induced depression‐ and anxiety‐like behaviors. (a) Experimental procedure. After 5‐day adaptation period, mice were injected with artesunate (0, 5, 15 mg/kg) twice daily for 9 days. On day 10, saline or LPS was administered systemically (0 or .8 mg/kg; i.p.). From day 11 to 14, mice were given additional injections of artesunate (0, 5, 15 mg/kg) 30 min prior to each behavioral test. Pretreatment with artesunate had no effect on time spent in the center zone (b) in the OFT. Pretreatment with artesunate significantly blocked the prolonged latency to feeding in the NSF (c), increased immobility time (d) without affecting the latency to immobility time in the TST (e), increased floating time (f) and decreased latency to floating time in the FST (g), decreased source preference (h) and total intake in the SPT (i). *p < .05, **p < .01, **p < .001 as compared to VEH group, ^#^ *p* < .05, ^##^ *p* < .01, ^###^ *p* < .001 as compared to LPS group, *n* = 8–9 per group.

Figure 3a indicates a schematic timeline of the treatment and behavioral assays. Mice were randomly divided into four groups (*n* = 8–9 per group): (1) saline injected twice daily for 9 days and followed by vehicle injection group (VEH group); (2) saline injected twice daily for 9 days and followed by LPS injection group (LPS group); (3) 5 mg/kg artesunate‐treated twice daily for 9 days and followed by LPS injection group (ART 5 mg/kg group); (4) 15 mg/kg artesunate‐treated twice daily for 9 days and followed by LPS injection group (ART 15 mg/kg group). On day 10, mice were injected with LPS and later were subjected to behavioral testing. Before each test, mice received additional injections of artesunate, as indicated in Figure 3a.

Figure 3b shows that during the OFT, mice treated with artesunate/LPS spent a similar amount of time in the center zone as LPS‐treated control mice. A one‐way ANOVA confirmed this observation and revealed no significant difference in the time spent in the center zone between groups (*F*_3,25_ = 0.372, *p* = .774, Figure 3b). These results indicate that artesunate pretreatment failed to block LPS‐induced reduction in open field activity.

In the NSF test, the LPS‐treated mice showed suppressed eating behavior in a novel environment. Mice pretreated with artesunate showed shorter latency to feeding than the LPS‐treated control. This observation was confirmed by a one‐way ANOVA that revealed a significant difference in the latency to feeding among three groups (*F*_3,25_ = 3.720, *p* = .032, Figure 3c). A post hoc Tukey's test showed that 15 mg/kg of artesunate prolonged the immobility latency as compared to the LPS‐treated control group (*p* = .016). The results suggested that artesunate pretreatment blocked LPS‐induced novelty‐suppressed feeding.

In the TST, mice treated with LPS showed significant greater immobility than the control (Figure 3d). Reduced immobility (time being motionless) was observed in mice pretreated with artesunate (Figure 3d). A one‐way ANOVA of the TST data showed there was a significant difference in the total immobility time among groups (*F*_3,25_ = 4.919, *p* = .028; Figure 3d). The latency to immobility time was not different between groups (*F*_3,25_ = 0.258, *p* = .885; Figure 3e). A post hoc analysis showed that the LPS treatment increased immobility time (*p* = .005) and this effect was prevented by 5 mg/kg of artesunate treatment (*p* = .041). The results indicate that artesunate pretreatment blocked LPS‐induced immobility in the TST.

In the FST, mice treated with LPS showed significant longer immobility time (floating time) than the vehicle‐treated mice (Figure 3f). However, when mice were pretreated with artesunate, their immobility time was drastically reduced (Figure 3f). A one‐way ANOVA revealed a significant artesunate treatment effect (*F*_3,25_ = 17.887, *p* < .001). A post hoc Tukey's test showed that LPS led to a longer floating time (*p* < .001) and this effect was prevented by both 5 mg/kg (*p* < .001) and 15 mg/kg (*p* < .001) of artesunate pretreatment. A separate ANOVA revealed that latencies to floating initiation were significantly different between groups (*F*_3,25_ = 4.346, *p* = .014; Figure 3g). Post hoc Tukey's test revealed that 5 mg/kg (*p* = .018) and 15 mg/kg (*p* = .007) of artesunate prolonged the immobility latency time as compared to the LPS‐treated group (Figure 3g). The results suggest that artesunate pretreatment blocked LPS‐induced immobility in the FST.

Figure 3h shows that LPS‐treated mice showed a significant reduction in sucrose preference as compared to the control. However, mice pretreated with artesunate acquired preference for sucrose (Figure 3h). A one‐way ANOVA confirmed this observation and revealed a significant difference in sucrose preference rate between groups (*F*_3,25_ = 3.664, *p* = .033). Post hoc Tukey's test revealed that the LPS administration decreased sucrose preference (*p* = .044), but pretreatment with 5 mg/kg (*p* = .041) and 15 mg/kg (*p* = .029) of artesunate prevented this deficit. Interestingly, the LPS or artesunate treatment did not affect total food intake as indicated in Figure 3i and by a one‐way ANOVA (*F*_3,25_ = 2.915, *p* = .054; Figure 3i). The results indicate that artesunate pretreatment blocked LPS‐induced immobility in the SPT.

Overall, these results indicate that pretreatment with artesunate has prophylactic potential and can prevent the anxiety‐ and depression‐like phenotypes caused by the LPS treatment.

## 4 DISCUSSION

In the present study, we investigated the neuroprotective potential of artesunate. Using the MTS cell proliferation assay, we found that artesunate on its own did not affect cellular activity, but pretreatment with artesunate significantly reduced H_2_O_2_‐induced cellular injury in PC12 cells, suggesting that artesunate exerts neuroprotective effects against cellular oxidative stress. In addition, we found that pretreatment with artesunate blocked behavioral deficits caused by the LPS treatment. Specifically, artesunate blocked LPS‐induced novelty‐suppressed feeding, evidenced as shorter latencies to initiation of food consumption in a novel environment. In the TST, the artesunate pretreatment reduced LPS‐induced immobility but had no effect on latency to immobility. In the FST, mice treated with LPS showed significant longer immobility time (floating time) than the vehicle‐treated mice, but not when pretreated with artesunate. Pretreatment with artesunate reduced floating time and latencies to floating initiation during the FST. In the SPT, mice treated with LPS showed a significant reduction in sucrose preference, but those pretreated with artesunate showed no LPS‐induced deficits and were able to develop a strong preference for sucrose. Last, artesunate did not affect spontaneous locomotor activity in the presence of LPS treatment. These findings are the first demonstration that artesunate can prevent LPS‐induced depression‐ and anxiety‐like symptoms, strongly suggesting its prophylactic potential in the treatment of depression.

Major depression is a chronic disorder whose symptoms can interfere with daily functioning and largely contribute to mental disabilities. Emerging evidence suggests that inflammation and oxidative stress are significant contributors to major depression (Porter & O'Connor, 2022; Smith, 1991; Yirmiya, 2000). Patients suffering from depression have high rates of inflammation in the central nervous system (Beurel et al., 2020; Dowlati et al., 2010; Yirmiya, 2000), and experimentally induced immune activation can lead to depression‐like behaviors in rodents (Zuo et al., 2014, 2016). Interestingly, anti‐inflammatory medications can be used to prevent or reduce depressive symptoms in patients suffering from major depressive disorder (Akhondzadeh et al., 2009; Muller et al., 2006) or in rodents exerting depression‐like symptoms (Zhang et al., 2019). In addition, chemogenetic silencing of IL‐1β, proinflammation cytokine that is highly active during oxidative stress and depression, significantly attenuates anxiety‐ and depression‐like behaviors in mice exposed to LPS (Li et al., 2017).

In the present study, we demonstrated that mice exposed to LPS showed depression‐like behaviors, evidenced as increased immobility during a swim test, reduced latency to feeding, and loss of sucrose preference. These LPS‐induced behavioral deficits were prevented by the artesunate pretreatment, suggesting that artesunate has prophylactic potential in the treatment of depression. To our knowledge, the derivatives of artemisinin also include dihydroartemisinin and artemether besides artesunate; however, we did not focus on them due to the poor water‐solubility and low bioavailability of dihydroartemisinin and artemether. We are hopeful that medication discovery research will focus on improving the structure and chemical properties of these compounds to address the existing issues.

These results are noteworthy for several reasons. First, current antidepression medications have limited efficacy and up to 30% of patients report no significant improvement in their mental state. Although antidepressants offer benefits to most patients suffering from depression, important problems persists such as intolerability, delayed therapeutic onset, side effects, and the existence of treatment‐resistant depression (Penn & Tracy, 2012). Thus, the wealth of research with tricyclic medications and selective serotonin reuptake inhibitors expanded our understanding of depression treatment and calls for more effective medications with a faster onset and fewer side effects. In addition, prophylactic treatments of mental health are gaining more public attention. In the search for such medications, we focused on artesunate, the FDA‐approved and WHO‐recommended medication that is commonly used to treat malaria (Dondorp et al., 2005). A number of clinical studies evaluated artesunate for its adverse events. Most common side effects of artesunate are dizziness, loss of appetite, nausea, and diarrhea (and less common liver damage or miscarriage) (Clark et al., 2008; White et al., 2006). Artesunate produces no serious psychiatric adverse events (Aneja et al., 2019; Sirima et al., 2016). In line with this, a recent study found that treatment of malaria with artesunate reduced neurologic deficits and reduced inflammation in children (Conroy et al., 2021). With an intravenous administration, artesunate is relatively safe and the CDC recommends using intravenous artesunate to treat severe malaria, including in pregnant women and children (Brejt & Golightly, 2019; Lalloo et al., 2016). Given its prophylactic potential and safety prolife, artesunate appears to be an excellent candidate for the NIH drug repurposing program, and certainly, it is worthy of further investigation.

Second, we have found that artesunate prevented H_2_O_2_‐induced oxidative stress and cellular injury in the PC12 cell line. These findings align with previous reports that in vitro artesunate treatment can prevent LPS‐induced inflammation by interfering with NF‐кB and p38 MAPK signaling (Mancuso et al., 2021; Okorji & Olajide, 2014). Furthermore, several studies report that the action of artesunate correlates with autophagy (Efferth, 2017; Jiang et al., 2018; Sun et al., 2019). Specifically, artesunate accelerates autophagy via suppression of the PI3K/AKT/mTOR signaling pathway (Feng & Qiu, 2018). Given that the PI3K/AKT/mTOR pathway plays a role in the pathophysiology of depression (Shi et al., 2012), it is conceivable that artesunate suppresses depressive‐like symptoms by suppressing this pathway. Artesunate is also known for its ability to downregulate the expression of cytokines such as p‐IκB‐α, IFN‐γ, IL‐17, and TNF‐α that are activated by the LPS treatment (Yang et al., 2012). Interestingly, high levels of inflammation produced by the same cytokines have been observed in patients suffering from major depression (Beurel et al., 2020; Dowlati et al., 2010; Yirmiya, 2000). Based on a strong correlation between inflammation and depression, we hypothesized that artesunate exerts antidepressant potential by suppressing oxidative stress. Our experiment was divided into two parts. The first part was the preliminary detection of anti‐inflammatory activity of artesunate through cell culture. The results showed that a low dose of artesunate significantly attenuated H_2_O_2_‐induced decrease in cell viability. The second part was to explore whether artesunate could prevent LPS‐induced depression‐ and anxiety‐like behaviors. The results from this experiment showed that artesunate significantly blocked the LPS‐induced depressive and anxiety‐like behaviors, further confirming the antidepressant and anxiolytic effects of artesunate.

Thus, our findings suggest that artesunate has neuroprotective potential and can produce antidepressant and anxiolytic effects, most likely by suppressing neuroinflammation. If so, potential application of artesunate for the treatment of depression and other CNS disorders affected by inflammation warrants further investigation.

## AUTHOR CONTRIBUTIONS

Shihao Huang, Ewa Galaj, Jinfeng Wang, Li Meng, and Haishui Shi designed the experiment; Shihao Huang, Jinfeng Wang, Yi Guo, Shuang Wang, Mengxu Shi, and Xueyong Yin conducted the behavioral experiments and Ewa Galaj wrote the manuscript; Xueyong Yin, Shihao Huang, and Li Meng analyzed the data; Ewa Galaj, Yixiao Luo, Shuang Wang, and Keyao Liu revised the manuscript.

## CONFLICT OF INTEREST

All authors declare no conflict of interest.

### PEER REVIEW

The peer review history for this article is available at https://publons.com/publon/10.1002/brb3.2833.

## Data Availability

All data generated for this study are included in the article.
